# Improved amyloid burden quantification with nonspecific estimates using deep learning

**DOI:** 10.1007/s00259-020-05131-z

**Published:** 2021-01-07

**Authors:** Haohui Liu, Ying-Hwey Nai, Francis Saridin, Tomotaka Tanaka, Jim O’ Doherty, Saima Hilal, Bibek Gyanwali, Christopher P. Chen, Edward G. Robins, Anthonin Reilhac

**Affiliations:** 1 Raffles Institution, Singapore, Singapore; 2 Present Address: Clinical Imaging Research Centre, Yong Loo Lin School of Medicine, National University of Singapore, Centre for Translational Medicine (MD6), 14 Medical Drive, #B1-01, Singapore, 117599 Singapore; 3 Memory Aging and Cognition Centre, National University Health System, Singapore, Singapore; 4 Department of Neurology, National Cerebral and Cardiovascular Center, Osaka, Japan; 5 Saw Swee Hock School of Public Health, National University of Singapore, Singapore, Singapore; 6 Department of Pharmacology, Yong Loo Lin School of Medicine, National University of Singapore, Singapore, Singapore; 7 Singapore BioImaging Consortium (SBIC), Agency for Science, Technology and Research (A*Star), Singapore, Singapore

**Keywords:** Alzheimer’s disease, Amyloid, Positron emission tomography (PET), Quantification, Deep learning, Nonspecific uptake

## Abstract

**Purpose:**

Standardized uptake value ratio (SUVr) used to quantify amyloid-β burden from amyloid-PET scans can be biased by variations in the tracer’s nonspecific (NS) binding caused by the presence of cerebrovascular disease (CeVD). In this work, we propose a novel amyloid-PET quantification approach that harnesses the intermodal image translation capability of convolutional networks to remove this undesirable source of variability.

**Methods:**

Paired MR and PET images exhibiting very low specific uptake were selected from a Singaporean amyloid-PET study involving 172 participants with different severities of CeVD. Two convolutional neural networks (CNN), ScaleNet and HighRes3DNet, and one conditional generative adversarial network (cGAN) were trained to map structural MR to NS PET images. NS estimates generated for all subjects using the most promising network were then subtracted from SUVr images to determine specific amyloid load only (SAβ_L_). Associations of SAβ_L_ with various cognitive and functional test scores were then computed and compared to results using conventional SUVr.

**Results:**

Multimodal ScaleNet outperformed other networks in predicting the NS content in cortical gray matter with a mean relative error below 2%. Compared to SUVr, SAβ_L_ showed increased association with cognitive and functional test scores by up to 67%.

**Conclusion:**

Removing the undesirable NS uptake from the amyloid load measurement is possible using deep learning and substantially improves its accuracy. This novel analysis approach opens a new window of opportunity for improved data modeling in Alzheimer’s disease and for other neurodegenerative diseases that utilize PET imaging.

**Supplementary Information:**

The online version contains supplementary material available at 10.1007/s00259-020-05131-z.

## Introduction

Globally, dementia is a leading cause of death with a doubling in prevalence from 1990 to 2016 [1]. Alzheimer’s disease (AD), the most common cause of dementia, is defined by abnormal deposits of amyloid-β (Aβ) plaques and neurofibrillary tau tangles in the brain [2]. Aβ plaques are the earliest detectable pathological biomarker and can develop 20 years before the onset of dementia [3]. Due to the increasing burden of AD, methods for early detection and hence prevention of AD have become increasingly critical. Early detection of Aβ deposition can be achieved using positron emission tomography (PET), which enables the in vivo examination of the spatial distribution and quantitative accumulation of Aβ plaques using amyloid-targeting radiotracers.

Currently, Aβ is generally quantified from static PET scans using the standardized uptake values ratio (SUVr) calculated by normalizing Aβ-PET tracer uptake with a reference region known to be devoid of Aβ [4]. However, Aβ radiotracers also bind nonspecifically to myelin protein in the white matter (WM), which also has β-sheet structure as the Aβ plaques [5, 6]. This nonspecific (NS) uptake in WM is subjected to global and local variations caused by factors such as age as well as the presence of cerebrovascular disease (CeVD) as detected by WM-hyperintensities (WMH), lacunes, cortical infarcts, and cerebral microbleeds (CMBs) [6, 7]. Aβ is typically quantified within the cortical gray matter (GM) regions, which also contains myelinated axons [8], leading to the contamination of unwanted NS signals with specific Aβ uptake. This contamination is further exacerbated by the limited spatial resolution capability of PET scanners and brain atrophy causing the WM signal to spill into the GM regions [9]. Semi-quantitative methods, such as SUVr, are unable to distinguish between specific binding to Aβ and NS binding to other proteins.

We have recently proposed a novel Aβ quantification method for static Aβ PET images yielding two computed biomarkers: Aβ_L_, which quantifies the global Aβ burden, and ns, which characterizes the NS uptake [7]. Both markers are obtained via modeling of the SUVr PET image in standardized MNI-space, as a linear combination of two template images derived from a pool of subjects, describing the amyloid deposition pattern and the NS binding. With this modeling approach, ns captures the global sources of intersubject NS variability allowing Aβ_L_ to better reflect the Aβ burden. However, local variations caused by CeVD, inaccuracies in spatial registrations and normalization between the subjects’ Aβ-PET images, and the use of generic templates for the modeling may bias the fitting process and affect the quantification of specific Aβ binding.

Recently, deep neural networks have been applied successfully in image-to-image translation, particularly for improving image quality or converting structural information of one modality to another such as translating magnetic resonance imaging (MRI) to computed tomography (CT) images [10–13], or from one MRI modality to another [14, 15]. In contrast, translation from structural MRI or CT to PET is still a largely unexplored area as PET conveys functional or protein density information which is to a certain extent unrelated to the structural or tissue contrast information provided by MRI or CT. This work builds upon the hypothesis that structural MRI contains the necessary information about the myelin content, WM condition, and localized manifestations of cortical infarcts, lacunes, and CMBs, enabling the use of deep learning-based mapping to estimate the subject’s NS radiotracer uptake. In particular, we envisioned that deep learning models will be able to map from the structural MRI the reduced NS uptakes observed in regions with WHM (high FLAIR signal and low T1w signal), lacunes, and cortical infarcts (low FLAIR and T1w signal).

Specific uptake images, from which a novel Aβ-PET marker is computed, are then simply obtained by subtracting the subject’s estimated NS map from the SUVr image, thus simultaneously removing the need for linear fit in MNI-space. In this manuscript, we first report comparative performance obtained with three different deep learning networks for the estimation of NS uptake maps. We then demonstrate the superiority of this novel Aβ PET marker over traditional SUVr analysis and Aβ load computation based on generic templates using an Asian cohort of 172 participants featuring a wide range of cognitive impairment, many of whom have significant CeVD.

## Materials and methods

### Participants, neuropsychological examination, and neuroimaging

A total of 172 participants featuring a wide range of cognitive impairment with varying severities of CeVD burden were recruited from the Memory Aging and Cognition Centre (MACC) at the National University of Singapore. Ethics approval was obtained from the National-Healthcare Group Domain-Specific Review Board in Singapore and the study was conducted in accordance with the Declaration of Helsinki, with written informed consent from all participants or accompanying relatives in their preferred language.

Cognitive function assessment of each participant was conducted using a locally modified version of the Mini-Mental Status Examination (MMSE) [16], the Montreal Cognitive Assessment [17], clinical dementia rating (CDR) scale questionnaire, as well as the detailed neuropsychological battery [18]. The neuropsychological battery assesses the following domains: attention, language, visuoconstruction, visuomotor speed, verbal memory, visual memory, and executive function. Raw scores from each assessment were transformed into standardized *Z* scores using the mean and standard deviation of the whole study sample with non-cognitive impaired subjects as a reference [19].

T1-weighted and T2-weighted fluid-attenuated inversion recovery (FLAIR) MR images used for the definition of the regions of interest (ROIs) as well as for the estimation of the nonspecific uptake were acquired simultaneously on the PET/MR scanner with a 12-channel head receive coil with T1/TE/TR = 900/3.05/1950 ms. T1-weighted images were reconstructed into 256 × 256 × 176 matrices of 1 mm^3^ isotropic voxel size, while T2-weighted FLAIR images were reconstructed into 192 × 256 × 64 matrices with 1 × 1 × 2 mm^3^ voxel size. MR images for analysis and visual rating of CeVD were acquired using a 3 T Siemens Magnetom Trio, A Tim scanner (Siemens Healthcare GmbH), and a 32-channel head receive coil. The standardized neuroimaging protocol included 3D T1-weighted images, FLAIR, T2-weighted images, and susceptibility-weighted images (SWI).

All subjects underwent a 30-min PET scan on the 3 T Biograph mMR (Siemens Healthcare GmbH) at the Clinical Imaging Research Centre (CIRC), 40 min after injection of 370 (± 15%) MBq of [^11^C]PiB. Each PET listmode datum was motion-corrected using an in-house developed rebinner [20] and reconstructed into a 3D matrix of 344 × 344 × 127 with voxel size of 2.09 × 2.09 × 2.03 mm^3^. 3D ordinary Poisson ordered-subsets expectation maximization with resolution modeling using 3 iterations and 21 subsets was applied with all corrections.

All [^11^C]PiB-PET images were visually assessed by a junior PET image analysis researcher, a senior neuro-PET researcher, and a certified neurologist using the rainbow 16-bit scale on the Siemens Syngo platform (Siemens AG, Germany) in the transaxial plane and also in the sagittal and coronal planes when needed. Scans were visually interpreted as positive or negative based on 6 AD-specific regions: frontal lobe, parietal lobe, temporal lobe, anterior cingulate, precuneus/posterior cingulate [7].

### Nonspecific Aβ uptake image estimation using deep learning

For this image translational task, we compared two convolutional neural networks (CNNs)—monomodal HighRes3DNet [21] and multimodal ScaleNet [22] with our previously developed monomodal cGAN network [23]. The three models were trained to map structural MR to NS PET images using 40 Aβ-PET SUVr images that exhibited mainly NS uptake (selected based on their low SUVr). The Aβ-PET SUVr images were paired with their corresponding structural T1-weighted and T2-weighted FLAIR images during training. The monomodal cGAN network was trained using T2-weighted FLAIR as our preliminary results showed the superiority of this MRI sequence over T1-weighted images to predict NS PET images [23]. The performance of the monomodal HighRes3DNet was tested in this work with each sequence independently, while the multimodal ScaleNet was trained using both images. Detailed information on the network implementations, network configurations, and parameter optimization of HighRes3Dnet, ScaleNet, and cGAN can be found in supplemental material 1. SUVr PET images as well as the T2-weighted FLAIR were coregistered to the structural T1-weighted MR images using ANTS (version 1.9) [24]. These 40 subjects were split into 2 groups: 34 for network training and 6 for independent evaluation, whereby the groups were kept unchanged for fair comparison of the networks.

### Network performance evaluation

A total of four metrics were used to assess and compare the suitability of the model for this image translation task: mean squared error (MSE), structural similarity (SSIM), mean relative error, (MRE) and histogram intersection (HI).

MSE measures the average pixel-wise squared difference between the estimated image (X) and ground truth (Y):

1 $$ MSE\left(X,Y\right)=\frac{1}{n}\sum \limits_{i=1}^n{\left({X}_i-{Y}_i\right)}^2 $$

SSIM estimates the perceptual difference between two images using the mean (μ) and standard deviation (σ) of the generated image (X) and ground truth (Y). Two variables, c_1_ (= 0.01 L)^2^ and c_2_ (= 0.03 L)^2^, are included to stabilize the division with low denominator, with L being the dynamic range of the pixel values.

2 $$ SSIM\left(X,Y\right)=\frac{\left(2{\mu}_x{\mu}_y+{c}_1\right)\cdotp \left(2{\sigma}_{xy}+{c}_2\right)}{\left({\mu}_x^2+{\mu}_y^2+{c}_1\right)\cdotp \left({\sigma}_x^2+{\sigma}_y^2+{c}_2\right)} $$

MRE quantifies the relative error between the mean of the generated image (X) and ground truth (Y), with respect to the mean of the ground truth (Y). The mean of the absolute MRE values was then computed for all the test subjects.

3 $$ MRE\left(\%\right)=\frac{\frac{1}{n}{\sum}_{i=1}^n{Y}_i-\frac{1}{n}{\sum}_{i=1}^n{X}_i}{\frac{1}{n}{\sum}_{i=1}^n{Y}_i}\times 100 $$

HI measures the intersection of two histograms as follows:

$$ HI=H(X)H(Y)={\sum}_{i=1}^n\mathit{\min}\left({H}_i(X),{H}_i(Y)\right) $$

where *H*(*X*) and *H*(*Y*) are the normalized histograms of the generated image (*X*) and ground truth (*Y*) and *n* is the number of histogram bins.

These metrics were measured in the whole brain (WB) and in the cortical GM using MRI-derived masks for the 6 evaluation subjects during network optimization. The optimized HighRes3DNet and ScaleNet were compared and the best CNN and cGAN networks were then used to generate the NS images of all 172 subjects.

### Neuroimaging biomarkers

#### MR biomarkers

For each participant, cortical and lacunar infarcts were graded on T1-weighted, FLAIR, and T2-weighted images following the STRIVE criteria [25]. CMBs were graded on SWI sequences using the Brain Observer MicroBleed Scale [26]. WMH volume was quantified by automatic segmentation using the FLAIR sequence [27, 28]. Hippocampus volume, an MR marker for neurodegeneration, was derived using a model-based automated procedure (Free Surfer, v.5.1.0) on T1-weighted MR images [29].

#### Aβ*-*PET biomarkers

In this work, we propose a novel Aβ-PET biomarker, named hereafter SAβ_L_, that is obtained from the subtraction in native space of the subject’s NS uptake volume, estimated with the optimal network, from the SUVr volume. For performance comparison, global SUVr values, measured in native space using the parcellated MRI, and global Aβ load (Aβ_L_), measured in MNI-space, were derived following methods previously described [7] (supplementary material 2).

### Evaluation of Aβ-PET quantification

Spearman’s correlation analysis was conducted to evaluate the degree of association of each of the Aβ -PET markers and the NS estimates with MR markers of CeVD. The performance of the PET markers was compared using the strength and confidence of their associations with various neuropsychological test scores.

## Results

**Fig. 1:**
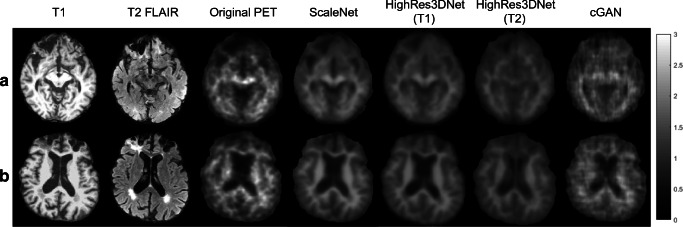
T1-weighted, T2-weighted FLAIR, and original PET images of a patient with (a) stroke and (b) WMH, with the corresponding NS images estimated using HighRes3DNet (T1-weighted and T2-weighted FLAIR input separately), ScaleNet, and cGAN (T2-weighted FLAIR as input). The PET and estimated NS images were set to the same image intensity range

Figure 1 shows the T1-weighted and T2-weighted FLAIR images of two subjects with the corresponding NS images estimated by HighRes3DNet, ScaleNet, and cGAN networks. These estimates can be directly compared with the corresponding actual PET images that mainly express NS uptake only. One subject has stroke and multiple CMBs (Fig. 1a) while the other has WMH and severe brain atrophy (Fig. 1b). cGAN produced estimates that visually looked the closest to the actual PET images, exhibiting similar textures and noise. ScaleNet produced uptake images with low noise and clear contrast between WM and GM. We note that the NS binding to central WM areas looks generally underestimated with all the tested networks.

Table 1 reports the performance evaluation of the optimized HighRes3DNet, ScaleNet and cGAN networks using MSE, SSIM, MRE, and HI averaged over the same six evaluation subjects. ScaleNet yielded the lowest MSE and highest image similarity in WB and GM as well as the smallest MRE in WB. cGAN yielded a slightly lower MRE in GM than ScaleNet. Also, cGAN estimates showed higher HI values in GM and WB, confirming the visual impression that it produces estimates with realistic noise and texture (see histograms of selected cases in Supplemental Fig. 2).

**Table 1:** Comparison of performance of HighRes3DNet, ScaleNet, and cGAN (mean ± stdev)

Metric	MSE		SSIM		MRE (%)		HI	
	WB	GM	WB	GM	WB	GM	WB	GM
HighRes3DNet (T1)	0.085 ± 0.017	0.056 ± 0.013	0.556 ± 0.029	0.600 ± 0.032	9.517 ± 2.233	8.275 ± 2.572	0.778 ± 0.038	0.676 ± 0.063
HighRes3DNet (T2)	0.126 ± 0.030	0.065 ± 0.016	0.463 ± 0.030	0.540 ± 0.031	12.512 ± 3.701	9.949 ± 4.072	0.750 ± 0.038	0.692 ± 0.048
ScaleNet	0.063 ± 0.009	0.042 ± 0.008	0.606 ± 0.035	0.659 ± 0.039	1.888 ± 2.916	1.977 ± 2.494	0.845 ± 0.044	0.743 ± 0.067
cGAN	0.106 ± 0.020	0.068 ± 0.009	0.399 ± 0.043	0.456 ± 0.042	3.372 ± 1.959	1.902 ± 2.477	0.877 ± 0.022	0.911 ± 0.023

**Fig. 2:**
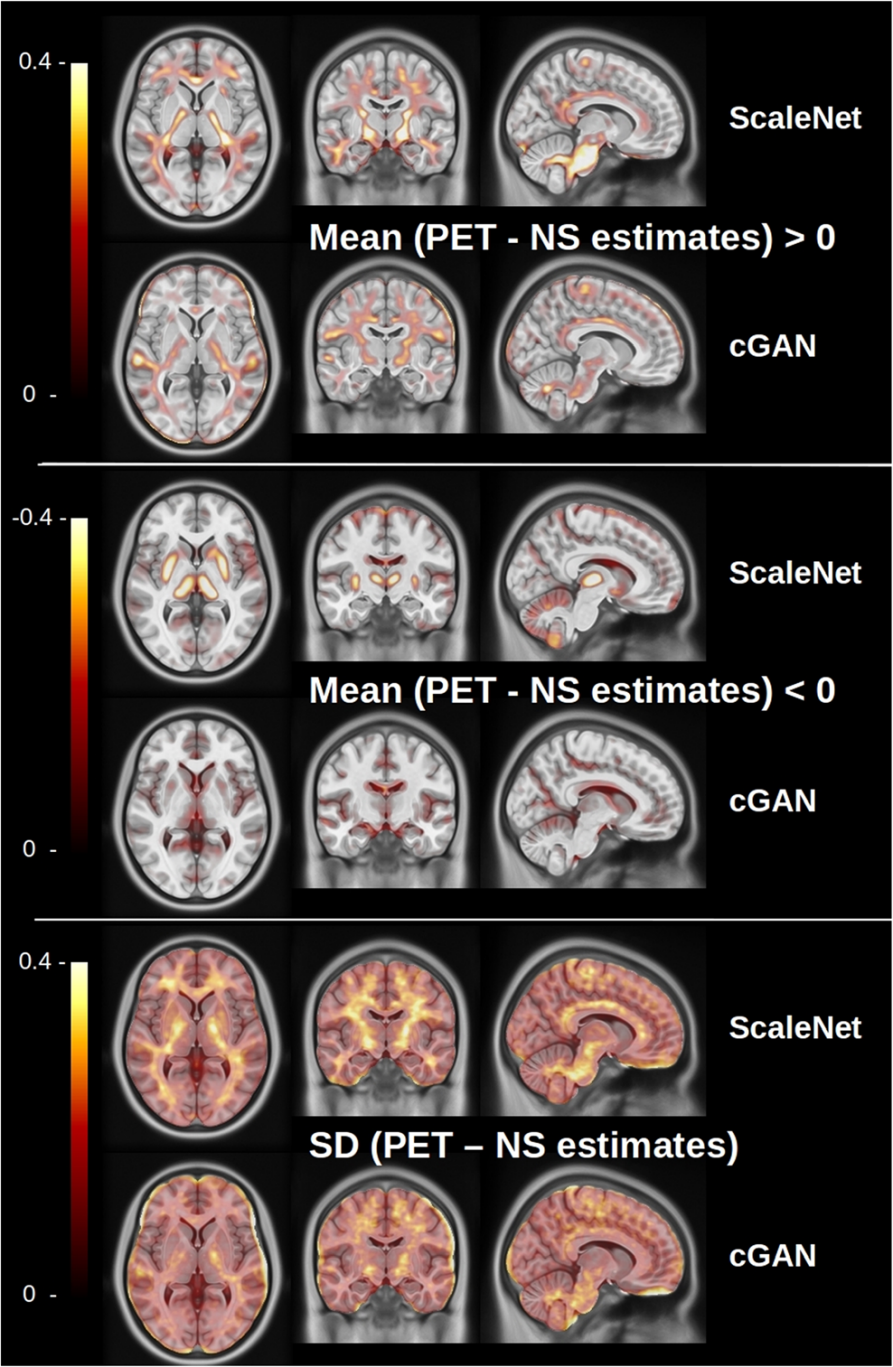
Mean SUVr absolute difference and standard deviation images computed in MNI-space from the 40 training PET scans and the corresponding NS images estimated with ScaleNet and cGAN. Top: regions where the NS is underestimated, middle: regions where the NS is overestimated, bottom: standard deviation images

HighRes3DNet using T1 or T2 images as input generated poor results with MRE in GM close to 10% and hence was removed from further evaluations. Figure 2 shows the mean absolute difference and standard deviation images computed in MNI-space from the 34 training and 6 evaluation PET scans and their corresponding NS images estimated with ScaleNet and cGAN. This closer look revealed that both ScaleNet and cGAN underestimated the NS binding for the training dataset in some central areas of WM. In addition, ScaleNet overestimated the NS uptake in the basal ganglia, the thalamus, and in the cerebellar GM.

NS estimates as well as specific uptake images SAβ_L_ were then generated with ScaleNet and cGAN using the remaining unseen scans from the whole cohort of subjects. This cohort included 21 subjects without CeVD (mean SUVr = 1.50 ± 0.51) and 151 subjects with significant CeVD (mean SUVr = 1.44 ± 0.43). Out of these 151 subjects, 77.5% of these cases had either WMH lesions (25.8%) or CMBs (6.6%) alone or presence of multiple CeVD markers (45%). The complete repartition is given in supplementary Table 3.

**Fig. 3:**
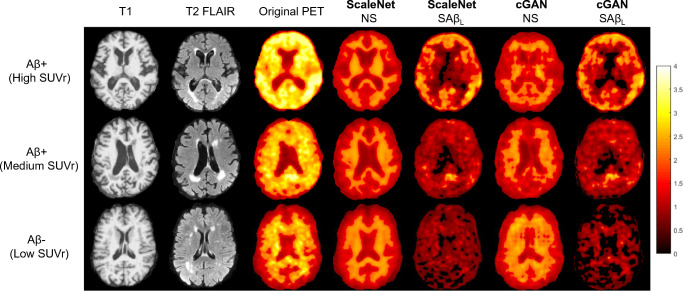
T1-weighted, T2-weighted FLAIR, and original PET images of patients visually classified as Aβ+, Aβ +, and Aβ− with high (top), medium (middle), and low (bottom) SUVr values. The NS images estimated from ScaleNet and cGAN, as well as derived SAβ_L_ volumes, are also shown. The PET and estimated images were set to the same image intensity range of 0–4 in hot-metal color-scale

Figure 3 shows, for 3 selected patients with different levels of Aβ burden, the structural MRI, corresponding SUVr images, NS estimates generated with cGAN and ScaleNet, and the resulting specific uptake images SAβ_L_ obtained from the subtraction of NS estimates from the SUVr images. We can observe that the underestimation of the NS binding in some WM areas led to residual NS signal in the SAβ_L_ images.

**Fig. 4:**
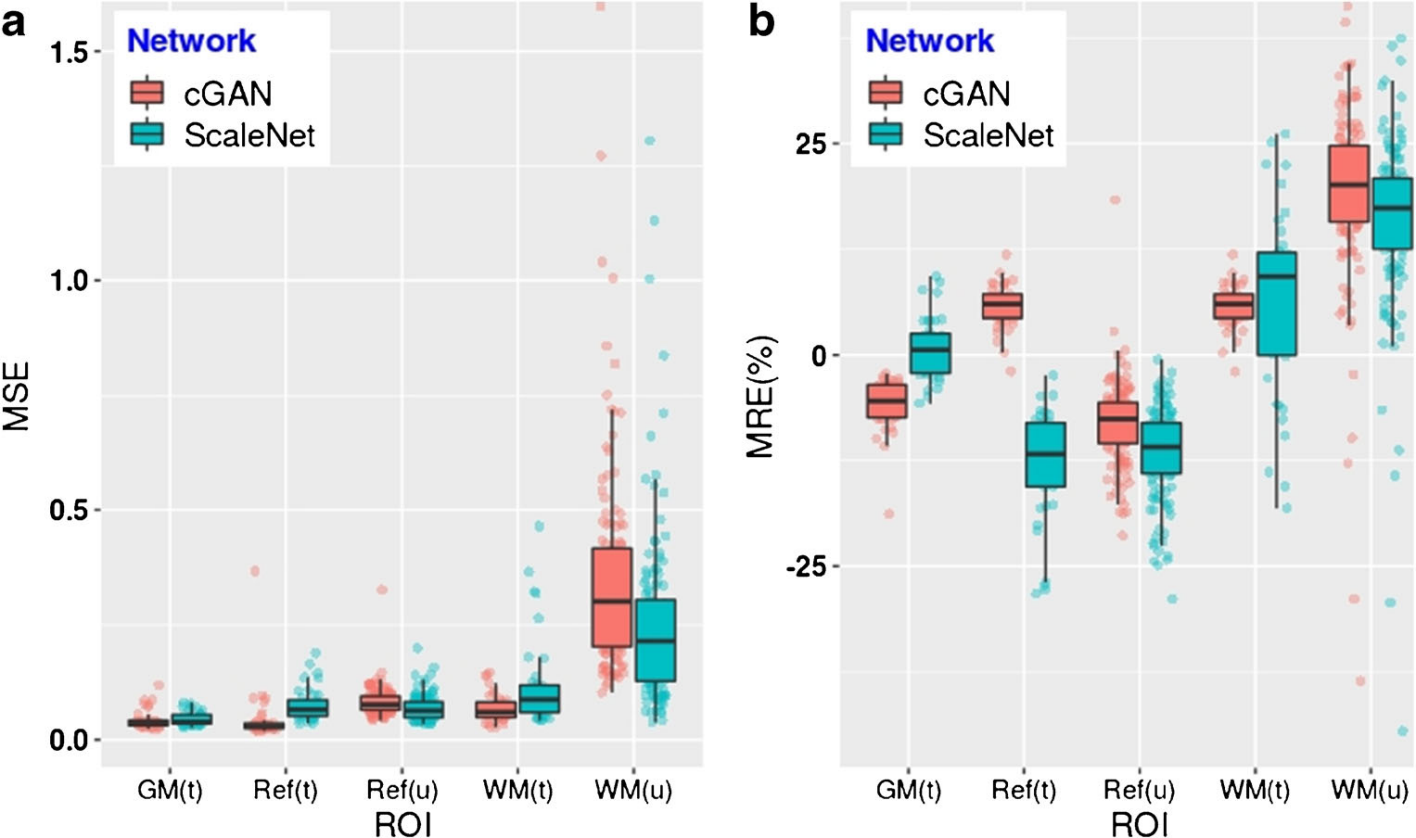
**a** MSE and **b** MRE (%) computed between actual PET scans and the NS estimated with ScaleNet (green) and cGAN (red) in the GM, WM, and reference region. (t) refers to measurements obtained from the 40 scans used for training and evaluation, while (u) refers to measurements obtained from 132 remaining unseen scans. ROI for WM was eroded to avoid contamination from specific uptake from the GM

Figure 4 provides quantitative indications on the extent of the inaccuracies in the central WM region and the cerebellar GM. Unlike with the training and evaluation data, cGAN overestimated the NS in the cerebellar GM for the unseen scans to the same levels as ScaleNet, which remained unchanged, indicating possible overfitting of the cGAN model. MRE in WM increased for the unseen data with both cGAN and ScaleNet. However, a closer look at the training and unseen scans revealed that NS uptakes in the WM matter of the unseen scans were on average 12.6% higher than of the training scans (see discussion). All these inaccuracies do not affect the Aβ quantification, with MRE, in the cortical GM, which is the only measurement that has an impact on the accuracy of the specific uptake in GM. MSE and MRE for GM are only shown for the training scans as most of the unseen scans contain specific binding to Aβ plaques. During the training phase, ScaleNet produced more accurate NS estimates for this critical region than cGAN with a MRE of 0.82% ± 3.65% compared to − 5.85% ± 3.09%.

**Fig. 5:**
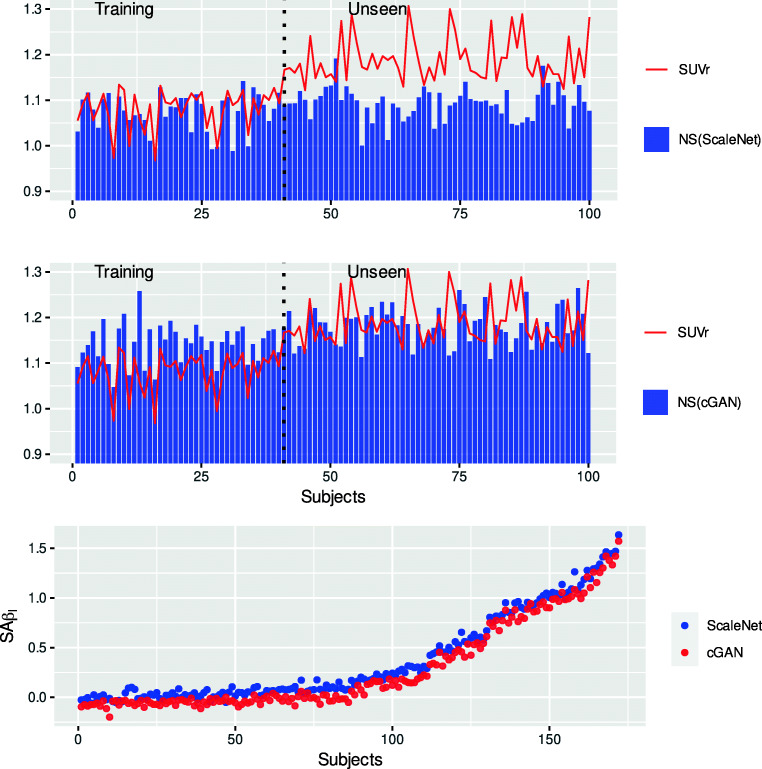
(Top and middle) NS measured in the GM from the ScaleNet and cGAN estimates of 100 subjects (40 training and 60 unseen) with low SUVr (< 1.3). The red line gives the corresponding SUVr values measured from the actual PET scans. (Bottom) Specific activity computed for the whole cohort using the NS estimates generated by ScaleNet and cGAN. Subjects were ordered on the X-axis with increasing SUVr values

Figure 5 shows the NS in the GM measured from the ScaleNet (top) and cGAN (bottom) estimates for the 100 subjects with the lowest SUVr (< 1.3). The red line gives the corresponding SUVr values measured from the actual PET scans. Note that the training dataset was built from the first 40 subjects. On the training section of the graph, ScaleNet produced more accurate NS estimates in GM than cGAN as shown in Fig. 4b. More interestingly, NS estimated by both networks show some differences but seem to follow the intersubject variations of the SUVr observed within the training datasets. These scans were selected for training purposes based on the assumption that they contained no or little Aβ and that intersubject variations were mainly driven by NS variations caused by CeVD. Observable differences can be attributed to model inaccuracies, but also possibly due to non-zero specific binding (scans were selected based on their SUVr not on their SAβ_L_, which was unknown then). NS estimates in GM obtained by ScaleNet for the unseen scans showed the same levels and magnitude of intersubject variations than with the training scans demonstrating the generalization of the models (i.e., no overfitting). However, in this case, intersubject variations of the SUVr were likely caused by variations of both specific and NS, making the direct comparison with the NS estimates less relevant. The bottom plot shows the SAβ_L_ values computed for the whole cohort using the NS estimates generated by ScaleNet and cGAN.

Table 2 reports the association of SUVr, NS, and SAβ_L_ with MRI-derived markers for CeVD and neurodegeneration. Three populations were studied: the whole cohort (top), the subset presenting no CeVD (middle), and the subset with low Aβ level (SUVr < 1.2). The results suggested that an increase of CeVD burden (number of cortical infarcts and lacunes) was associated with a reduction of the Aβ level when measured with the classical SUVr. The negative impact of CeVD on SUVr was even stronger in the subset with low Aβ. Contrary to cGAN, ScaleNet generated NS estimates showing the same negative associations with the various CeVD markers. SUVr was also positively correlated with atrophy in the whole cohort. This was expected as Aβ is very likely one explanatory factor of neurodegeneration. The association was stronger in the subset with no CeVD in which other likely sources of neurodegeneration are discarded. However, a negative association was found between SUVr and atrophy in the group with low SUVr values. The new biomarker, SAβ_L_(SN), computed from the subtraction of the ScaleNet’s NS images to the SUVr images did not show any associations with CeVD. In addition, the new biomarker showed a substantial increase in the positive association with neurodegeneration in groups containing Aβ + subjects while, contrary to SUVr, no association was found in the subset containing Aβ- subjects only, a group in which Aβ should not play a role. Finally, most of these unwanted associations remained with SAβ_L_(cGAN) due to lower associations of the cGAN NS estimates with CeVD and atrophy than the ScaleNet estimates.

**Table 2:** Association of PET amyloid markers and NS estimates with MRI-derived markers of CeVD and neurodegeneration (Spearman ρ, *p* value < 0.1; * < 0.05; ** < 0.01, *** < 0.001)

All subjects (*n* = 172)	SUVr	NS (SN)	SAβ_L_(SN)	NS (cGAN)	SAβ_L_(cGAN)
Number of CMBs	− 0.02	− 0.09	0.02	− 0.15*	0.02
Number of cortical infarcts	− 0.17*	− 0.22**	− 0.11	− 0.08	− 0.15 .
Number of lacunes	− 0.18*	− 0.25***	− 0.12	− 0.15.	− 0.13 .
WMH	4.58E-03	− 0.2**	4.79E-02	− 0.09	0.02
Hippocampal volume	− 0.38***	0.3***	− 0.43***	0.13 .	− 0.38***
Cortical atrophy grade	0.10	− 0.47***	0.19*	− 0.20**	0.13 .
No CeVD (*n* = 21)					
Hippocampal volume	− 0.67**	0.53*	− 0.69***	0.33	− 0.71***
Cortical atrophy grade	0.43 *	− 0.79***	0.44*	− 0.59**	0.44*
SUVr < 1.2 (*n* = 82)					
Number of CMBs	− 0.12	0.034	− 0.14	− 0.11	− 0.02
Number of cortical infarcts	− 0.33**	− 0.32**	− 0.13	− 0.12	− 0.27*
Number of lacunes	− 0.37***	− 0.39***	− 0.13	− 0.23*	− 0.18 .
WMH	− 0.12	− 0.20 .	0.04	− 0.05	− 0.09
Hippocampal volume	0.19.	0.34**	− 0.02	0.06	0.22*
Cortical atrophy grade	− 0.26*	− 0.51***	0.06	− 0.11	− 0.21 .

**Fig. 6:**
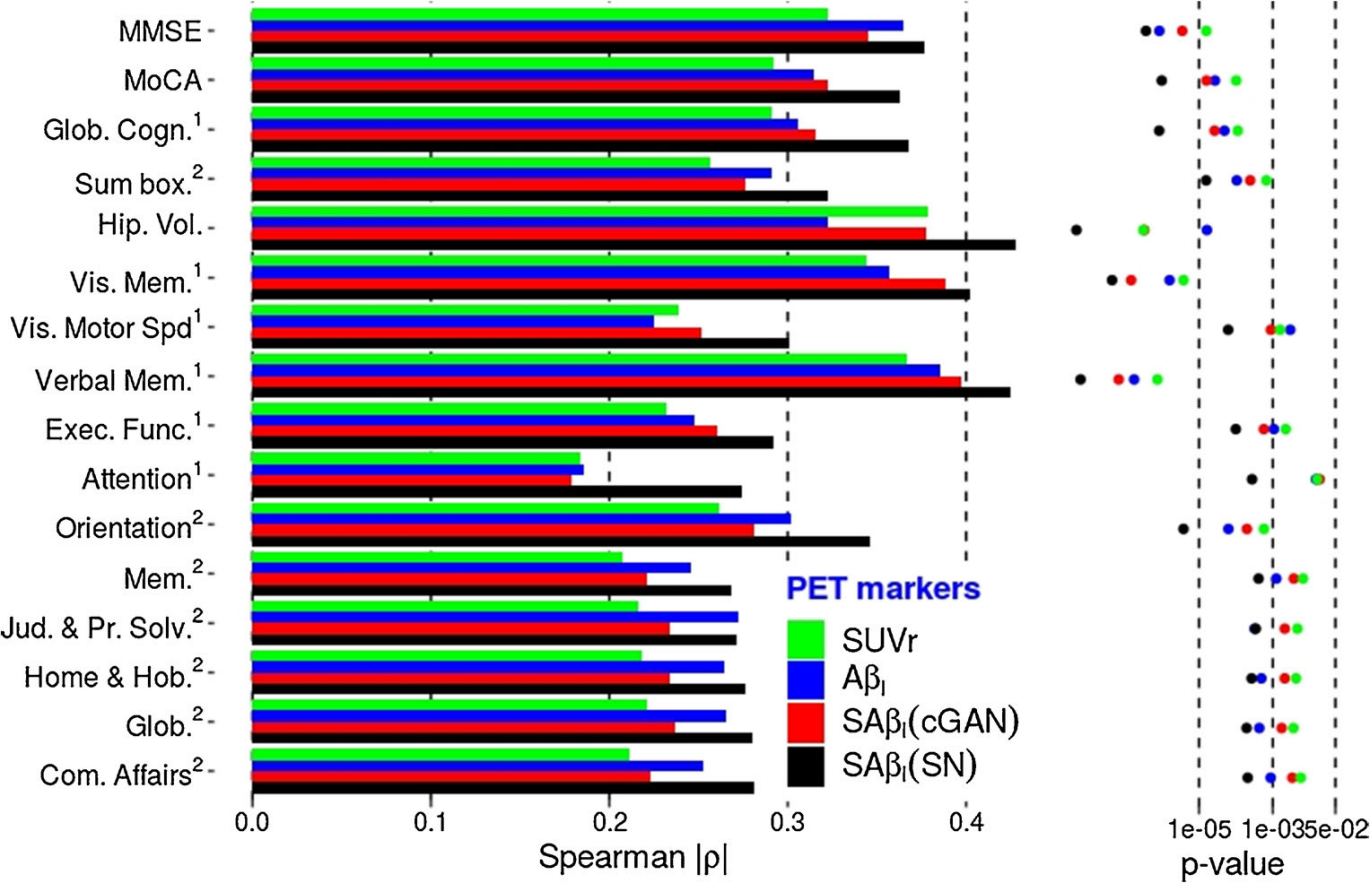
Association (Spearman’s *ρ*) and confidence (*p* values) of 4 tested PET biomarkers of the brain Aβ burden with cognition and neurodegeneration using the whole cohort (*n* = 172). The novel biomarkers SAβ_L_ (cGAN) and SAβ_L_ (SN) can be compared with the standard approach using SUVr measured in MRI-space, or with Aβ_L_, the previously proposed Aβ load computed in MNI-space using generic NS and specific templates

Figure 6 shows that compared to SUVr and Aβ_L_, substantially higher associations and confidence were obtained between SAβ_L_(SN), the novel amyloid marker computed using the ScaleNet estimates, and cognitive scores as well as neurodegeneration markers, with increase in Spearman ρ coefficients ranging from 13 to 49%. Association increases by up to 67% were observed when the analysis was limited to subjects with CeVD (see Supplemental Fig. 3). However, the average association increase was 4.69% only when the analysis was limited to subjects with moderate to high level of amyloid (see Supplemental Fig. 4). SAβ_L_(cGAN) followed the same trend, but with lower *ρ* values and confidence than with Saβ_L_(SN).

## Discussion

Current methods to quantify Aβ burden from Aβ-PET scans can be sensitive to variations of the tracer’s NS binding to the myelin that influenced by the presence of CeVD. In this work, we proposed a novel Aβ-PET quantification approach that harnesses the intermodal image translation capability of convolutional networks to remove this undesirable source of contamination and variability.

Neural networks were rarely applied in structural MRI to PET image translation tasks compared to other image modality translation such as from MRI to CT [10–13] due to the more complex—and very often non-existent relationship between the information conveyed by both modalities. Most published works on MRI to PET translation used models to produce pseudo [^18^F]FDG-PET images with cortical uptake variations driven by structural features such as cortical thickness and gyrus morphology [30–33]. Closer to our subject, Wei et al. [34] proposed a multimodal Sketcher-Refiner GAN to capture the relationship between various sequences of diffusion tensor imaging as well as of T1-weighted MR images and the myelin map obtained with [^11^C]PiB PET in multiple sclerosis patients. Contrary to most published works where neural networks are trained to directly produce the image of interest, in this work, we used deep learning networks to estimate, from the subject’s structural MRI, its corresponding Aβ- PET image, showing only the NS binding of the Aβ tracer and its variation due to different CeVD markers. This NS binding presents an unwanted source of contamination and variability to the measurement of Aβ level. In order to produce images of the specific Aβ-binding only, we proposed to remove this NS binding from the actual Aβ-PET image using the NS estimate. This is conceptually close to the work of Yaakub et al. [31] in which pseudo-normal [^18^F]FDG-PET images of epileptic patients were generated to improve the detection of epileptogenic zones from the actual [^18^F]FDG-PET images. Our hypothesis in this work was that convolutional networks are able to learn the complex mapping from myelin content and CeVD manifestation in MRI to the NS uptake image of the Aβ tracers. Results showed that out of the 3 tested networks, multimodal ScaleNet using both T1-weighted and T2-weighted FLAIR sequences as inputs was the most suitable candidate for this multimodal translation task. The numbers of training and evaluation scans were limited by the number of Aβ- scans in the cohort. We decided to select the first 40 scans with the lowest SUVr (< 1.13), leaving 34 scans for training and only 6 for evaluation. Note that this selection criterion was based on the measured SUVr values, the markers we aimed to challenge with the new marker. A retroactive analysis demonstrated that some of the scans that were selected for training contained some level of Aβ according to their corresponding SAβ_L_. Nevertheless, our results showed that during the training phase, ScaleNet produced NS estimates with MRE (%) below 1% in the GM. When the model was applied to the 6 evaluation scans that it had not been exposed to during the training, the MRE (%) in GM of the resulting estimates remained below 2%. We also verified that NS estimates in GM for the remaining 132 unseen (Aβ+) scans were of the same level and contained the same magnitude of intersubject variations as the training scans. More importantly, NS estimated with ScaleNet conveyed the same variations as in SUVr, with significant negative associations with the number of cortical infarcts, lacunes, and WMH grade. Once NS was subtracted from the SUVr, and contrary to the latter marker, the resulting novel biomarker SAβ_L_(SN) did not show any association with CeVD. NS also showed strong negative associations with markers of neurodegeneration: cortical and hippocampal atrophies (i.e., positive association with hippocampal volume). The removal of NS to SUVr increased the positive association of SAβ_L_(SN) with these markers. In the subset of subjects with low SUVr (< 1.2), the subtraction of the NS corrected the contradictory situation in which higher atrophy was associated with lower SUVr. Finally, the novel biomarker SAβ_L_(SN) showed increased monotonic associations with cognitive test scores by up to 67% compared to the conventional biomarker, meaning the new ranking of the scans based on their increasing SAβ_L_(SN) values is more associated with reduced cognitive and functional test scores. The removal of the NS can be seen as an efficient spillover correction that not only removes the source of contamination and variability originating from the WM but also the NS binding in the GM. As a matter of fact, no attempt was made in this work to correct the data for partial volume effects as most correction approaches rely on having a segmented MRI spatially registered with the PET image, and segmentation methods do not always perform well in the presence of CeVD [7]. In addition, these methods often assume a homogeneous activity uptake within each region, an assumption that would be often violated with the manifestation of CeVD. Our approach does not require the segmentation of the MRI. While this was not observed with our cohort, we however suspect its accuracy depends on the quality of the MR images and their spatial registration with the PET images, and visual inspection for artifacts and spatial mismatches should be performed beforehand.

Overall, CeVD and atrophy play a detrimental role in the accuracy of SUVr measurement and leads to its dependence on these conditions. Independence of predictor variables is an important criterion in data modeling in general, including machine learning-based methods for diagnosis and disease trajectory prediction. Linear regression is often used in mixed-type cohorts to investigate the joint effect of CeVD and Aβ burden on neurodegeneration and cognition. In a side analysis, we found that the dependence of SUVr on CeVD led to the following aberrations using the subset with low Aβ load: linear regression with MMSE as the outcome, age, SUVr, and number of cortical infarcts independent variables showed SUVr as a significant predictor (*p* < 0.05), with an unintuitive positive effect while the number of cortical infarcts was not. Replacing SUVr by SAβ_L_(SN) led to a significant negative effect (*p* < 0.05) of the number of cortical infarcts on cognition and no effect of Aβ load in this group where it should not play any role. This dependence could also have a detrimental effect in longitudinal studies in which longitudinal variations of the Aβ and CeVD markers are used to explain cognitive decline. In situations where the actual Aβ burden remained unchanged, any increase of CeVD burden and atrophy over the duration of the study would lead to a decline of the measured subject’s SUVr.

Our previous attempt to reduce the NS contribution and variation to the Aβ burden measurement relied on the modeling in the standardized MNI-space of the SUVr as a linear combination of 2 templates representing the NS specific and Aβ deposition pattern [7]. This approach has advantages over the original implementation proposed by Whittington et al. [35] in that the templates are derived from a cross-sectional study using principal component analysis in lieu of parametric modeling of longitudinal Aβ PET data. Our newly proposed novel biomarker, SAβ_L_, outperformed Aβ_L_ in association analyses with cognitive and functional tests when the cohort consisted of all the subjects or when it was limited to subjects with CeVD. However, Aβ_L_ showed higher associations when the analyses were performed on subjects with moderate to high amyloid levels only (supplemental Fig. 4). Aβ_L_ and SAβ_L_ computations follow very different processes. For instance, our SAβ_L_ implementation uses reference and target regions from the subject’s parcellated MRI to compute first the SUVr image and then to measure in native space the mean cortical SAβ_L_, while Aβ_L_ computation uses generic regions defined in MNI space. Segmentation of the cortical gray matter from structural MRI of subjects with CeVD or with severe atrophy can be challenging and inaccurate. Further investigations are required to find definite answers, but measuring the mean cortical SAβ_L_ from the SAβ_L_ images in MNI space is conceptually feasible and will be tested in future developments.

The three networks underestimated the NS uptake in some central WM areas (Fig. 2 top) however without having a possible impact on the Aβ quantification in the GM. Additional analyses were conducted to understand the cause (see Supplementary Fig. 4). They revealed that the training dataset contained mostly PET scans with low NS binding (SUVr below 2), while most of the unseen scans exhibit higher NS binding. The reason behind this difference in NS binding in these central areas between training and unseen Aβ-PET scans is still unclear and will require further investigations. The limited number of training scans may partially explain this inhomogeneous repartition. The small sample size of the whole cohort (*n* = 172) definitively constitutes a limitation of this study. The training dataset was built using the 40 scans with the lowest amyloid levels. Increasing this number would expose the networks during the training phase with scans with higher specific amyloid uptakes. Inter-subject variations of the level of specific binding are unpredictable from the structural MRI. We suppose that in order to minimize the RMSE, networks such as ScaleNet produce estimates with a small positive offset that is constant across subjects and which corresponds to the average specific uptake across training data. This may explain why ScaleNet overestimates the NS in the reference region as this region does not contain specific uptake. Further investigations need to be done, however, if this compensation mechanism is demonstrated; this mean offset could be deduced using estimated NS uptakes in the reference region and removed globally from the NS estimates.

Finally, Aβ-PET radiotracers, such as [^18^F]Amyvid, [^18^F]Neuraceq, [^18^F]Vizamyl, [^18^F]Flutafluranol, and [^11^C]PiB, are known to differ from each other by their levels of NS and specific binding, making direct quantitative comparisons impossible. Removing the NS binding using networks specifically trained for each of these tracers and then normalizing the resulting specific images to a common scale (i.e., 0%–100%) could pave the way for optimal data harmonization in multicenter studies using various Aβ-PET tracers.

In conclusion, removing the undesirable NS uptake from the Aβ load measurement is possible using deep learning networks and substantially improves its accuracy. Multimodal ScaleNet outperformed other networks in predicting the NS content in cortical GM with a MRE below 2%. Compared to SUVr, the resulting Aβ load measurements with SAβ_L_ showed increases of up to 67% in its association with cognitive and functional test scores. This novel analysis approach paves the way for improved statistical analysis, data modeling, and data harmonization in AD, especially with CeVD with mixed pathological changes, and potentially for other neurodegenerative diseases that utilize PET imaging.

## Supplementary Information

ESM 1 (DOCX 951 kb).
