# Supplementary material for: Improved amyloid burden quantification with nonspecific estimates using deep learning
Source: Eur J Nucl Med Mol Imaging. 2021 Jan 7;48(6):1842–53. doi: 10.1007/s00259-020-05131-z (PMC8113180; doi:10.1007/s00259-020-05131-z)
Supplement: Supplementary file 1 — (DOCX 951 kb). [file 259_2020_5131_MOESM1_ESM.docx]

**Supplementary Materials**

European Journal of Nuclear Medicine and Molecular Imaging

**Improved Amyloid Burden Quantification with Nonspecific estimates using Deep Learning**

Haohui Liu^1^, Ying-Hwey Nai^2^, Francis Saridin^3^, Tomotaka Tanaka^4^, Jim O’ Doherty^2^, Saima Hilal^3,5^, Bibek Gyanwali^6^, Christopher P. Chen^3^, Edward G. Robins^2,7^, Anthonin Reilhac^2^

^1^ Raffles Institution, Singapore.

^2^ Clinical Imaging Research Centre, Yong Loo Lin School of Medicine, National University of Singapore, Singapore.

^3^ Memory Aging and Cognition Centre, National University Health System, Singapore

^4^ Department of Neurology, National Cerebral and Cardiovascular Center, Osaka, Japan

^5^ Saw Swee Hock School of Public Health, National University of Singapore, Singapore

^6^ Department of Pharmacology, Yong Loo Lin School of Medicine, National University of Singapore, Singapore

^7^ Singapore BioImaging Consortium, Agency for Science, Technology and Research (A*Star), Singapore.

**Corresponding author:**

Ying-Hwey Nai

Address (Current): Clinical Imaging Research Centre, Yong Loo Lin School of Medicine, National University of Singapore, Centre for Translational Medicine (MD6), 14 Medical Drive, #B1-01, Singapore 117599, Singapore.

E-mail: [mednyh@nus.edu.sg](mailto:mednyh@nus.edu.sg), [yinghweynai@yahoo.com](mailto:yinghweynai@yahoo.com)

Tel: +65 65167412

Fax: +65 6872 0458

**Supplementary Material 1**

*Convolutional Neural Network (CNN)*

Two types of CNN were implemented and tested: monomodal HighRes3DNet [1] and multimodal ScaleNet [2]. HighRes3DNet utilises 3D-dilated convolutions to compute features with high spatial resolution as well as residual connections. The network consists of 20 layers of convolutions in total, in which the first seven use 3 × 3 × 3-voxel convolutions to capture low-level image features such as edges and corners. The next six layers contain kernels dilated by a factor of two, followed by six layers with kernels dilated by a factor of four. These twelve layers encode mid- and high-level image features. Residual connections [3-4], which enable direct information propagation from any residual block to another while bypassing the parameterised layers in a network to increase training efficiency are incorporated. Within each residual block, batch normalisation is first performed followed by the activation, which is tuned to find the most optimal activation function, followed by the convolution. In the last layer, 1 × 1 × 1-voxel convolutions were used followed by softmax to produce the generated PET volume. Multimodal ScaleNet uses HighRes3DNet as the frontend and uses a modality-dependent backend, with scalable layers that allows multi-input sharing without losing their original content. The merging layer, which acts as the interface between the backend and frontend, uses averaging to make the frontend structurally independent of the backend, thus allowing the multimodal ScaleNet to be more scalable.

These two networks are available on NiftyNet (Version 0.5.0) [5], a TensorFlow-based convolutional network platform, which uses patch-based sampling of the image data. The networks were kept unchanged for ease of comparison with other works, but the network parameters were optimized with about 30 different hyperparameter configurations (<https://niftynet.readthedocs.io/en/dev/config_spec.html>) within the computational feasibility of the CPU (Dell OptiPlex 9020). The networks were trained using 250 iterations as determined from Tensorboard where the loss function curve has flattened within each network, beyond which the improvement was small or insignificant. A spatial window patch size of (256,24,24) was used for both HighRes3DNet and ScaleNet, while a batch size of 13 and 16 was employed for HighRes3DNet and ScaleNet respectively. Weighted window sampling, linear interpolation, PReLU (Parametric Rectified Linear Units) activation and Adam optimiser were employed with B-spline post-smoothing. The training objective of both networks is to reduce the root mean squared error (RMSE) between the generated PET image and the real PET image. The details of both networks’ configuration can be found in Supplementary Table 1.

**Supplementary Table 1**: Details of network configurations of HighRes3DNet (T1), HighRes3Dnet (T2) and ScaleNet.

| Hyperparameters | HighRes3DNet (T1) | HighRes3DNet (T2) | ScaleNet |
| --- | --- | --- | --- |
| Activation function | prelu | prelu | prelu |
| Optimizers | Adam | Adam | Adam |
| Batch size | 16 | 16 | 16 |
| Input Interpolation | Linear (1) | Linear (1) | Linear (1) |
| Learning rate | 0.005 | 0.005 | 0.005 |
| Loss function | RMSE | RMSE | RMSE |
| Whitening | False | True | False |
| Normalization | False | True | False |
| Regularization type | L2 | L2 | L2 |
| Sample per volume | 8 | 8 | 10 |
| Volume padding size | 16 | 16 | (21, 21, 21) |
| Window sampling | weighted | weighted | weighted |
| Spatial window size | (256, 24, 24) | (256, 24, 24) | (256, 24, 24) |
| Inference window size | (256, 48, 48) | (256, 48, 48) | (256, 48, 48) |
| Output Interpolation | B-spline (3) | B-spline (3) | B-spline (3) |

*Conditional Generative Adversarial Network (cGAN)*

The implementation of cGAN and reported preliminary results were described elsewhere, with the optimised cGAN obtained using T2-weighted FLAIR images as input [6]. cGAN is coded in Keras and learns the mapping from an input MR image *x*, with a random noise vector *z*, to output PET image *G(x)*, *G: {x, z} → G(x)* [7]. The generator G is trained to produce realistic PET images from the input MR image that cannot be distinguished from real PET images. Concurrently, the adversarially-trained discriminator D is trained to detect the generator’s produced PET images and differentiate between the generated and real PET images. The objective of G is to not only reduce the error between the generated PET image and the real PET image, and to maximise the likelihood of the discriminator D classifying the generated image as “real”. The Adam optimizer is used for both D and G, with a batch size of 1, Gaussian weight initialization, label smoothing, two-time scale update rule (TTUR) [8] and spectral normalization [9]. Additional preprocessing of the 3D MR and PET volumes are required for cGAN implementation, including slicing of 3D MR and PET volumes to 2D RGB images in the axial plane and taking 132 out of the 176 slices for training, and linear normalization of image data from [global minimum, global maximum + 0.5] to [-1, 1]. Similarly, the inverse was carried out for post-processing, after which the output images were smoothed using 2 mm Gaussian filter width using SPM (<https://www.fil.ion.ucl.ac.uk/spm/>).


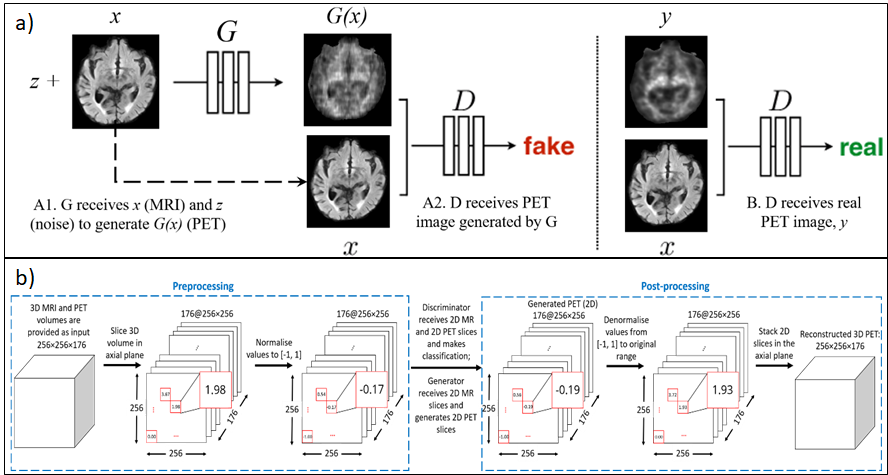


**Supplementary Fig. 1**: Overview of cGAN network: (a) cGAN training process and (b) pre- and post-processing of input and output images.

**Supplementary Table 2**: Details of network configurations of cGAN.

| Hyperparameters | cGAN (generator) | cGAN (discriminator) |
| --- | --- | --- |
| Activation function | tanh | sigmoid |
| Optimizers | Adam | |
| Batch size | 1 | |
| Weight Initialization | Gaussian (Mean = 0, Standard Deviation = 0.02) | |
| Learning rate | 0.0001 | 0.0004 |
| Loss function | L1+Binary Cross-Entropy | Binary Cross-Entropy |
| MRI Modality | T2-weighted | |
| Normalization | Preprocessing:  Linear from [minimum, maximum+0.5] to [-1, 1]  Post-processing:  Linear from [-1, 1] to [minimum, maximum+0.5] | |
| Spectral Normalization | True | |
| Label Smoothing | [0.7, 1.2] for real images  [0.0, 0.3] for generated images | |
| Slices taken | 132 out of 176 in the axial plane | |
| Spatial window size | (256, 256, 1) | |
| Inference window size | (256, 256, 1) | |
| Output Interpolation | 2mm Gaussian | |

**References**

1. Li W, Wang G, Fidon L, Ourselin S, Cardoso MJ, Vercauteren T. On the compactness, efficiency, and representation of 3D convolutional networks: brain parcellation as a pretext task. In: International conference on information processing in medical imaging. Springer, Cham; 2017. p. 348-60.
2. Fidon L, Li W, Garcia-Peraza-Herrera LC, Ekanayake J, Kitchen N, Ourselin S, Vercauteren T. Scalable multimodal convolutional networks for brain tumour segmentation. In: International Conference on Medical Image Computing and Computer-Assisted Intervention. Springer, Cham; 2017. p. 285-93.
3. He K, Zhang X, Ren S, Sun J. Deep residual learning for image recognition. In: Proceedings of the IEEE conference on computer vision and pattern recognition. IEEE; 2016. p. 770-8.
4. He K, Zhang X, Ren S, Sun J. Identity mappings in deep residual networks. In: European conference on computer vision. Springer, Cham; 2016. p. 630-45.
5. Gibson E, Li W, Sudre C, Fidon L, Shakir DI, Wang G, et al. NiftyNet: a deep-learning platform for medical imaging. Computer methods and programs in biomedicine. 2018 May 1;158:113-22. <https://doi.org/10.1016/j.cmpb.2018.01.025>
6. Liu H, Nai YH, Chen C, Reilhac A. Deep Learning-Based Estimation of Non-Specific Uptake in Amyloid-PET Images from Structural MRI for Improved Quantification and Diagnosis of Alzheimer's Disease. In: 2020 IEEE 33rd International Symposium on Computer-Based Medical Systems (CBMS). IEEE; 2020. p. 576-81.
7. Isola P, Zhu JY, Zhou T, Efros AA. Image-to-image translation with conditional adversarial networks. In: Proceedings of the IEEE conference on computer vision and pattern recognition. IEEE; 2017. p. 1125-34.
8. Heusel M, Ramsauer H, Unterthiner T, Nessler B, Hochreiter S. Gans trained by a two time-scale update rule converge to a local nash equilibrium. In: Proceedings of the 31st International Conference on Neural Information Processing Systems. Curran Associates Inc.; 2017. p. 6626-37.
9. Miyato T, Kataoka T, Koyama M, Yoshida Y. Spectral normalization for generative adversarial networks. arXiv preprint arXiv:1802.05957. 2018 Feb 16.

**Supplementary Material 2**

*Derivation of SUVr and AB_L_*

SUVr volumes were generated in the MRI native space using the cerebellar GM as the reference region. Global SUVr values were then measured using a cortical target encompassing 6 predefined Aβ-specific regions (orbitofrontal, frontal, posterior cingulate, parietal, temporal, occipital). Aβ_L_ values were obtained after spatial normalisation of the Aβ-PET images into the MNI-space. SUVr volumes were first generated using a generic reference ROI for the cerebellar GM. The global Aβ load (Aβ_L_) was then derived from the modeling of the SUVr volume as a linear combination of two template images describing the nonspecific binding component (NS) and the carrying capacity or the maximum possible concentration of Aβ (K) [1]:

| $SUVr=ns\times NS+{A\beta}_{L}\times K$ | (1) |
| --- | --- |

**References**

1. Tanaka T, Stephenson MC, Nai YH, Khor D, Saridin FN, Hilal S, et al. Improved quantification of amyloid burden and associated biomarker cut-off points: results from the first amyloid Singaporean cohort with overlapping cerebrovascular disease. Eur J Nucl Med Mol Imaging. 2020 Feb 1;47(2):319–31.


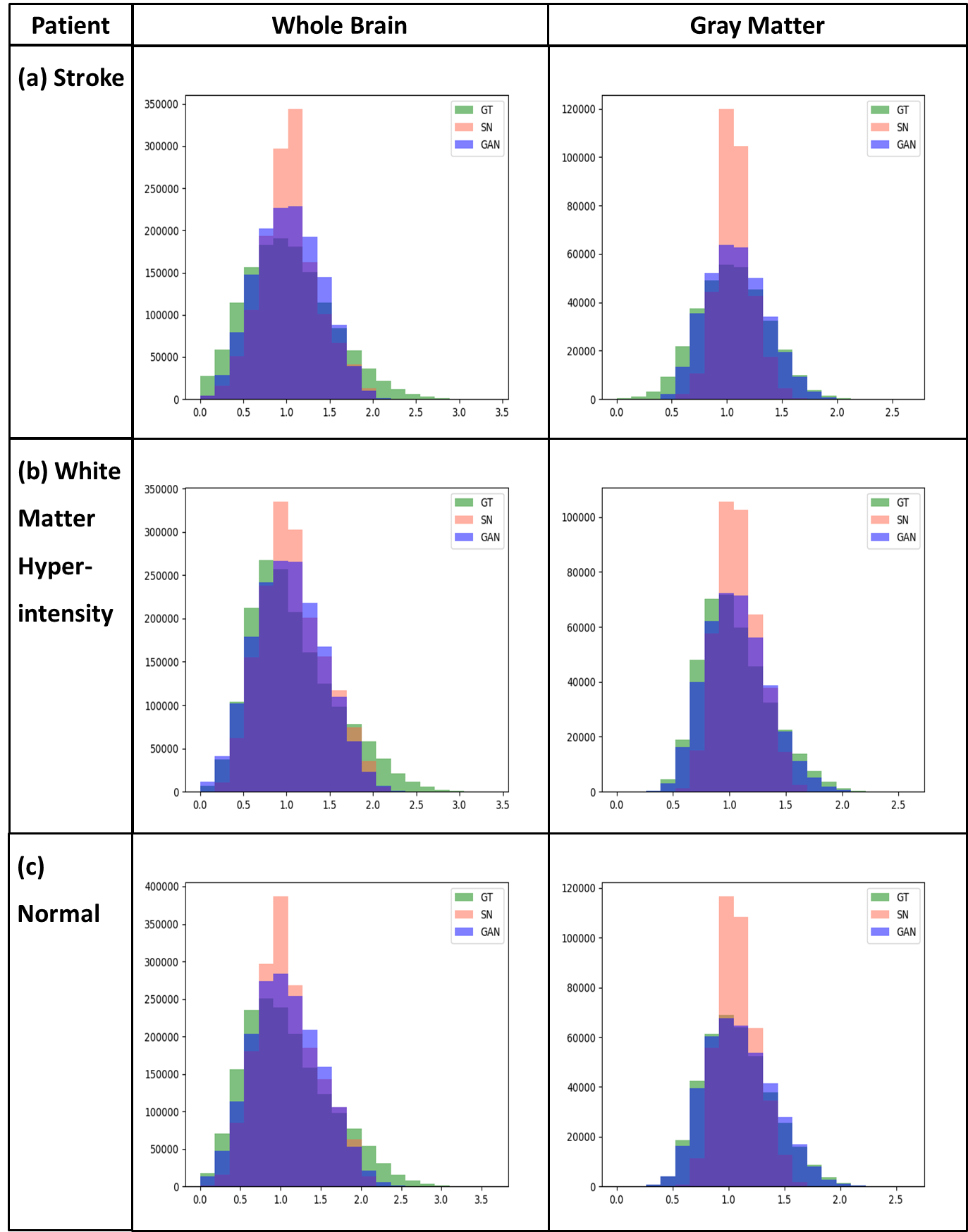


**Supplementary Fig. 2:** Histograms of ground truth image (GT, green) versus ScaleNet (SN, pink) and cGAN (purple) estimated images generated on three test subjects: (a) with stroke, (b) with white matter hyperintensity and (c) normal.


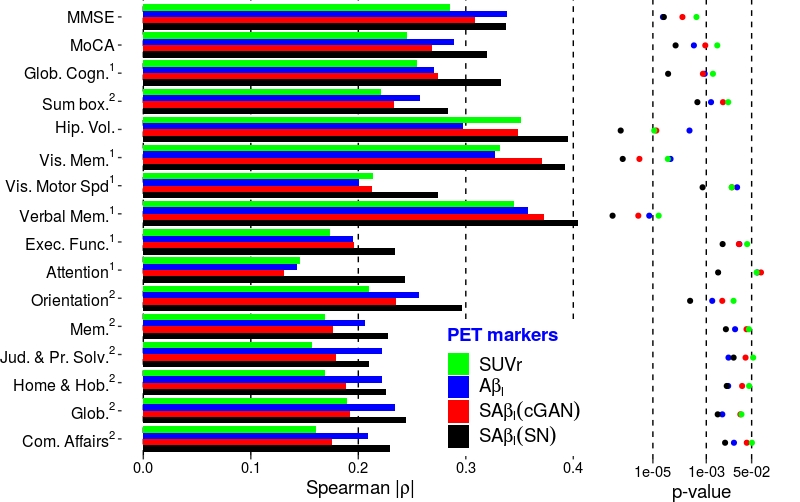


**Supplementary Fig. 3**: Association (Spearman’s ρ) and confidence (p-values) of 4 tested PET biomarkers of the brain Aβ burden with cognition and neurodegeneration using the subset of subjects with CeVD (n=81). The novel biomarkers SAβ_L_(cGAN) and SAβ_L_ (SN) can be compared with the standard approach using SUVr measured in MRI-space, or with Aβ_L_, the previously proposed Aβ load computed in MNI-space using generic NS and specific templates.


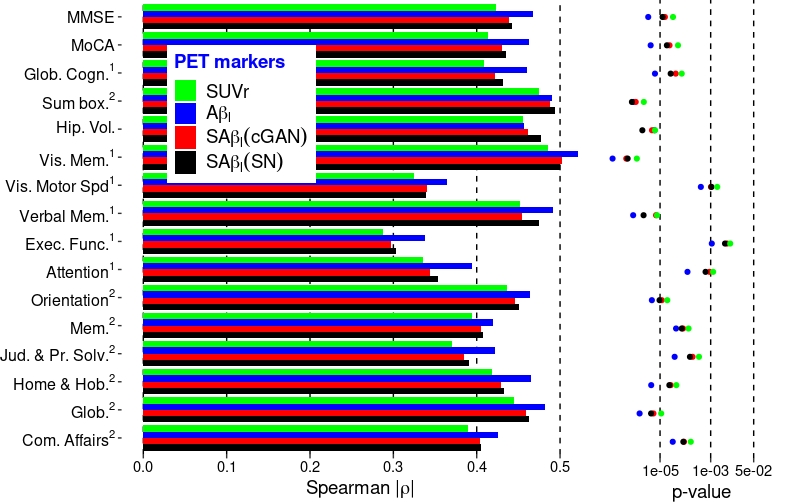
**Supplementary Fig. 4**: Association (Spearman’s ρ) and confidence (p-values) of 4 tested PET biomarkers of the brain Aβ burden with cognition and neurodegeneration using the subset of subjects with moderate to high level of amyloid (SUVr > 1.2, n = 90). The novel biomarkers SAβ_L_(cGAN) and SAβ_L_(SN) can be compared with the standard approach using SUVr measured in MRI-space, or with Aβ_L_, the previously proposed Aβ load computed in MNI-space using generic NS and specific templates.


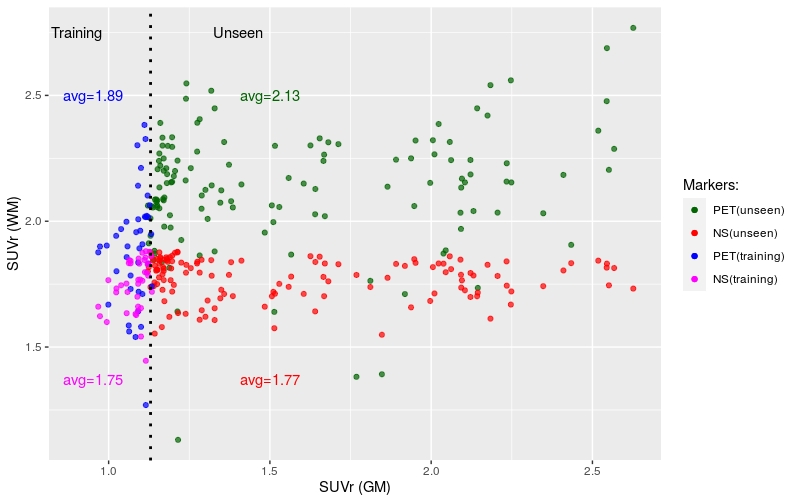


**Supplementary Fig. 5**: Mean SUVr measured in the central WM areas from the training and unseen original PET scans as well as from the NS estimates generated using the ScaleNet model. WM SUVr values are plotted as a function of the SUVr measured in the GM.

| CeVD | Total | WMH | CMBs | Cortical infarcts | Lacunes |
| --- | --- | --- | --- | --- | --- |
| WMH | 129 | 28.83 | 45.03 | 15.89 | 33.11 |
| CMBs | 84 | 45.03 | 6.62 | 11.92 | 27.15 |
| Cortical infarcts | 29 | 15.89 | 11.92 | 0 | 10.60 |
| Lacunes | 58 | 33.11 | 27.15 | 10.60 | 0.66 |

**Supplementary Table 3**: Number of subjects with each CeVD (Total) and their overlapping factors (%). For instance, out of the 151 subjects presenting CeVD, 28.83% present WMH lesions only and 45.03% concomitantly with CMBs.
